# Interprofessional Collaboration Improves Quality of Life of a Young Adult With Rett Syndrome

**DOI:** 10.7759/cureus.36921

**Published:** 2023-03-30

**Authors:** Jeri L Bullock, Katie Gradick, Craig Proctor, Marcy A Rogers, Wendy L Hobson

**Affiliations:** 1 School of Dentistry, University of Utah Health, Salt Lake City, USA; 2 Department of Pediatrics, University of Utah Health, Salt Lake City, USA

**Keywords:** caries, complex medical conditions, interprofessional care, chronic medical care, dentistry, rett syndrome

## Abstract

Rett syndrome (RTT) is a genetic neurodevelopmental disease characterized by early normal development followed by regression in motor and language skills. Patients with RTT often exhibit seizure disorders, growth failure, heart and lung disorders, bruxism, and dental caries. We report on a female patient in her 20s with Rett syndrome who presented to her primary care clinic with increasing agitation and pain. This case reports describes a collaborative, interprofessional approach between medical and dental providers who work in an integrated outpatient setting. It demonstrates that interprofessional collaboration, goals of care discussions, and attention to social drivers of health can improve quality of life for a medically and socially complex patient.

## Introduction

Rett syndrome (RTT) is a severe neurodevelopmental disease typically caused by sporadic mutations in Methyl-CpG-binding protein 2 (MECP2) that affects approximately one in 10,000 live female births [1]. Children with RTT often display normal development in the few months to a year of life and then begin to show regression of motor and language skills, as well as deceleration of head growth [2]. Additional manifestations of RTT include growth failure, epilepsy, breathing disorders, autonomic nervous system dysfunction, cardiac abnormalities, and sleep and behavioral disturbances [1,3].

Common oral health conditions associated with RTT include bruxism, gingivitis, and dental caries [4,5], with reported prevalence of bruxism estimated between 80-94% [6,7]. Despite these significant oral health needs, families of children with special health care needs, including RTT, are more likely than healthy children to face financial and dentist-related barriers to care [8,9]. Parents of children with special health needs must often prioritize multiple, urgent medical issues and navigate waitlists, with limited time and resources [9]. Parents of children with RTT have reported difficulty in finding a dentist who is willing to treat their child, due to behavioral disturbances and other special needs [9]. 

The profound cognitive impairment associated with RTT makes preventive home care difficult for caregivers, increasing the individual’s risk for caries and periodontal disease. Preventive treatments like fluoride varnish, and professional dental cleanings can reduce the risk of caries and periodontal disease but may be inaccessible for reasons discussed above. When dental treatment is necessary, a variety of therapeutic and pharmacological treatment methods are used in oral health treatment for patients with RTT including mouth props, analgesia for light or moderate sedation, and general anesthesia [4]. Dental extractions can be indicated for patients with RTT and have been found to be more common in households with low annual income, as in the general population [8].

Layered onto intrinsic dental issues, poor social drivers of oral health worsen outcomes for those on the lower end of the social gradient [10]. Dental caries and periodontal disease increase for those who live in poverty [11]. The World Health Organization’s Commission on Social Determinants of Health recommended health practitioners be aware of social drivers of health, to teach future generations about them, and to create programs to decrease inequities [12]. The issues related to social drivers of health are complex, and often more difficult to teach within the dental context [13], interprofessional collaborations in primary care clinics aimed at addressing these issues may be a way to improve oral health.

This case study will demonstrate the ways that interprofessional collaboration, goals of care discussions, and attention to social determinants of health can improve quality of life for a medically complex patient. An interprofessional team, including two primary care physicians, a care coordinator, two general dentists, three dental students, an anesthesiologist, and a surgeon, collaborated to provide care that aligned with the family’s goals.

## Case presentation

A young adult (in her 20s) with Rett syndrome presented to her primary care clinic with increased agitation and self-injurious behavior, concerning for untreated pain. The family reported extreme difficulty in performing daily dental hygiene for the patient due to biting. Her primary care clinic is a free standing, interprofessional teaching clinic comprised of dentists, midwives, physician assistants, nurse practitioners and family medicine, pediatric, and obstetric physicians. All clinicians work in the same hallway and utilize a common electronic health record; it functions as an integrated interprofessional clinic.

Relevant medical history for the patient included history of significant self-injurious behaviors, bowel obstruction, gastrostomy-jejunostomy (GJ) tube placement, cataracts, delayed development, epilepsy, feeding difficulties, weight loss, spasticity, poor dental hygiene, and behavioral disturbances that had resulted in injury to her caregivers. While the patient had a GJ tube, she continued to eat food by mouth and the GJ tube was used for venting. Significant aspects of the family’s social history included Spanish as their preferred language, not having citizenship in the United States, limited education, lacking dental insurance, and being medically underinsured and at times uninsured. Access to regular dental care was inhibited by lack of dental insurance, costs of care for sedation and surgical procedures, and long wait times for specialized dental care. Our interprofessional teaching clinic is comprised of dentists, midwives, physician assistants, nurse practitioners and family medicine, pediatric, and obstetric physicians. It is co-located in the same hallway and utilizes a common electronic health record.

On examination, the patient had visible caries and tooth erosion, as well as gingival erythema, prompting referral to the co-located dental team with concern for dental abscess. Identifying sources of pain in children with medical complexity can be difficult, particularly for those who cannot self-report symptoms [14]. For this patient, the primary medical team considered a broad differential, including menstrual cramping, constipation, subclinical seizures, acute otitis media, gastroesophageal reflux disease, sleep disturbance, as well as dental pain. Odontogenic sources of pain should always be considered in the differential diagnosis for these patients [14]. Care coordination between the referring primary care physician, the family, and the receiving dental team was essential to achieve successful out-patient service. The plan included a pre-procedural oral sedation administered by the physician, with an integrated visit scheduled for the medical and dental team to treat the patient synchronously.

The initial dental examination to assess the source of pain was performed in the out-patient dental clinic, without access to anesthesia support. The care team included a general dentist, three dental students and the referring primary care physician. Certified bilingual staff were present for the entire encounter. The role of the primary care physician was to administer the pre-procedure oral sedation and to monitor vitals during the procedure. A pre-procedure oral dose of five milligrams of diazepam 60 minutes prior to the procedure was not sufficient to achieve adequate sedation due to the patient’s chronic benzodiazepine use. An additional 10 milligrams of diazepam were administered 30 minutes before the procedure. The patient remained visibly agitated so the patient’s typical nighttime dose of 150 milligrams of quetiapine was administered, allowing the patient to be transferred to the dental chair for the examination. Nitrous oxide sedation was appropriately administered to a level of 30% N2O2 for a duration of 30 minutes. Oxygen saturation and heart rate were monitored continuously during the examination. Blood pressure was monitored before and after the examination. Dental student number one performed passive head stabilization, dental student number two placed and secured a mouth prop while dental student number three performed a dental exam. Attempts to obtain quality radiographs were hindered by patient movement. The dental clinical confirmed the differential diagnosis of a dental abscess associated with non-restorability on teeth number four, five and twenty-seven. Several other areas of demineralized enamel were noted, and sodium diamine fluoride (SDF) was applied to these areas to medically manage caries [15]. Due to the patient’s continued agitation during the exam, the dental team could not safely perform the necessary extractions in an out-patient setting and determined a referral for general anesthesia was required.

The coordinated plan for synchronous, interprofessional treatment accomplished its goal of identifying acute oral health needs in the patient. The oral and nitrous sedation allowed the patient to receive a dental exam in a safe, monitored setting. Having the dental and medical primary teams present in the same clinic and collaborating intensely instilled confidence in the patient’s family that her overall well-being is highest priority.

Coordination of care following the out-patient dental exam required the collaboration of multiple teams outside of the clinic. Deep sedation for patients with RTT is considered high-risk due to many unique features of RTT, which include episodic hyperventilation, breath holding, bloating and autonomic dysfunction [16]. The family’s primary language and limited medical insurance and lack of dental insurance were key factors in the decision process for coordinating care. Limiting the patient’s exposure to deep sedation was essential, and the care coordination team identified an upcoming gastrojejunostomy (GJ) tube exchange under general anesthesia as an opportunity to perform concurrent dental procedures. In exploring the family’s goals of care, it was clear that eating by mouth brought this patient joy and improved her quality of life. While they were eager to obtain adequate pain control with limited extraction, they did not wish to pursue full extraction, given the impact it would have on her ability to eat by mouth.

The medical surgical team agreed to allow the dental team to perform the treatment immediately after the medical procedure if the anesthesia team approved. The anesthesia team agreed to an additional time for the dental team to complete the necessary procedures. The operating room time was adjusted to accommodate the additional dental procedures and the medical and dental teams continued to communicate right up to the surgery day. The patient’s care coordinator assisted the family in completing the required pre-operative COVID-19 testing. The medical team completed an interventional radiology gastrostomy-jejunostomy tube (IR GJ) exchange followed by the dental team performing the dental work. Two of the original dental students and a general dentist with hospital privileges performed a dental exam, prophylaxis, and extractions of number four, five and twenty-seven. Both medical and dental procedures were completed without complications under general anesthesia. 

The primary care physician provided post-operative pain control recommendations. Due to a history of increased pain sensitivity and pain-related agitation, a longer opioid taper was utilized. Following her recovery from the procedure, the family reported that the patient was calmer and more comfortable than she had been in years. Her self-injurious behaviors had effectively resolved, her sleep improved, and she required fewer as-needed agitation medications.

**Figure 1:**
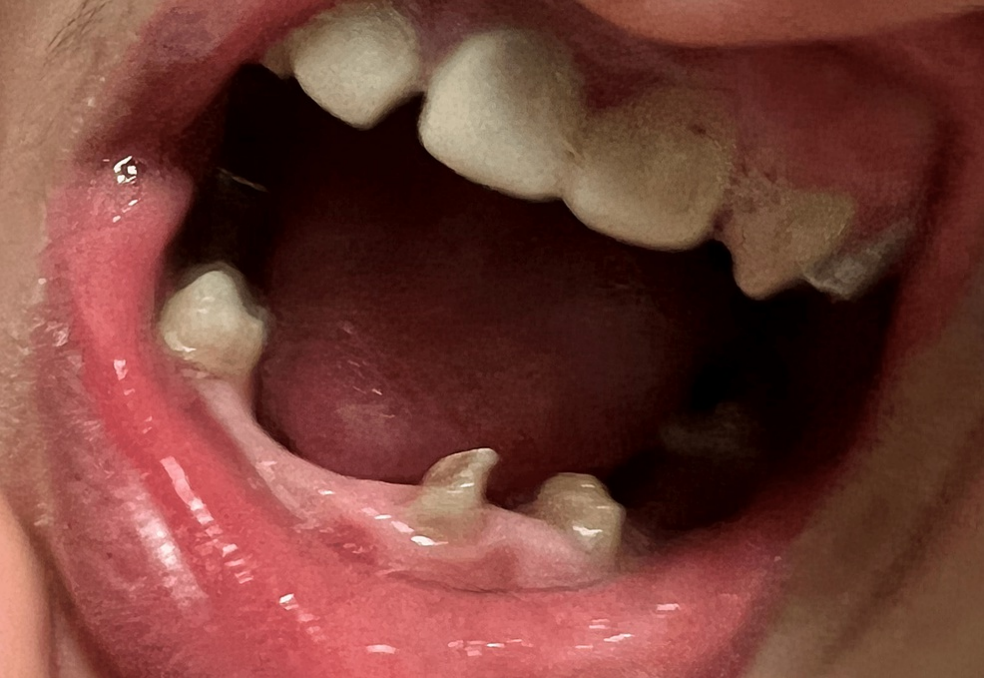
Oral exam in fall 2022, open view Photo of extraoral mouth exam demonstrating marked decay and erosion of teeth #6, 7, 21, 25, 28, 29, and 31. Photo taken at visit by physician. Guardian granted permission to share.

**Figure 2:**
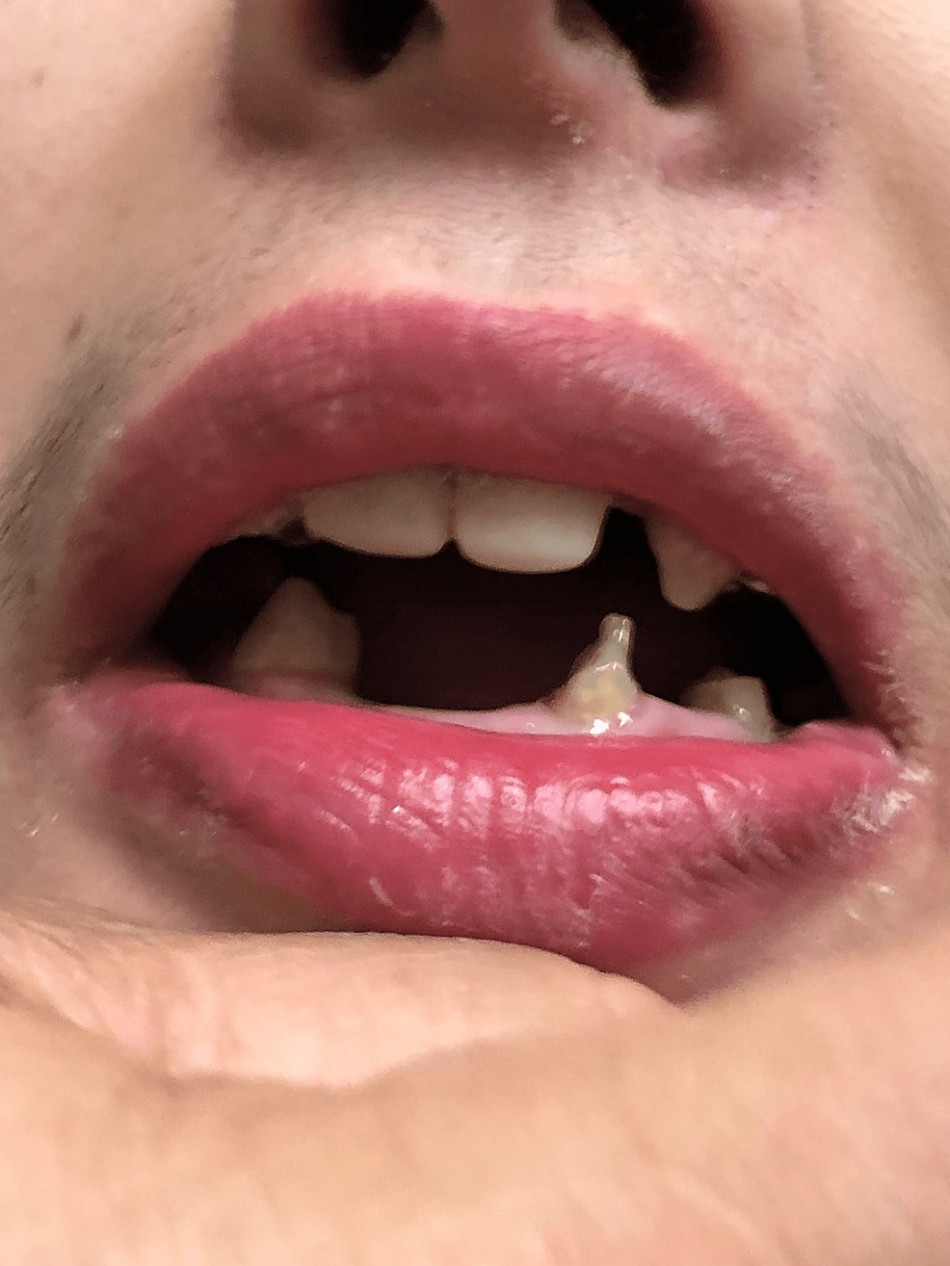
Oral exam in fall 2022 Photo of extraoral mouth exam demonstrating marked decay and erosion of tooth #21. Photo taken at visit by physician. Guardian granted permission to share.

Fifteen months later, the patient presented with increasing pain and agitation. The broad differential for her increased pain and agitation was reconsidered, and the family’s goals of care were re-explored. Her physical exam was normal for her except for her oral exam which revealed visible caries and tooth erosion, without significant gingival erythema (Figures 1, 2).

Given recurrence of pain and probable infection, the primary care physician and dentist discussed options with the family, including full extraction. So that the issue did not continue to recur annually, the family now believed that full extraction was the best option for the patient. She could continue to take liquids and purees by mouth, and a swallow study was planned to ensure that she did not have aspiration. To meet the needs of the patient and family, the interdisciplinary coordination of care team planned her treatment.

Before her scheduled dental surgery date, she was admitted to the hospital with an acute infection and seizures. The primary care team was contacted by the inpatient service. During the discussion of care, the family and team decided not to proceed with full extraction, and instead “that if a tooth were still "good" that we would leave it in place to help with some masticatory function.” She had the dental procedure in the operating room while having a gastrostomy tube placed. During the hospitalization the neurologist started her on levetiracetam and lacosamide for seizure disorder. 

In a follow-up virtual visit by her primary care provider, she still had some crying, which her mother attributed to upcoming menses. She was still enjoying eating and had no coughing with feeds; therefore the swallow study was cancelled.

Throughout her care, a bilingual medical care coordinator, who works closely with a dental care coordinator and the family was critical for communication. A detailed summary of the patient’s care timeline can be seen in Table 1.

**Table 1:** Summary of care timeline SDF: sodium diamine fluoride; IR GJ: interventional radiology gastrostomy-jejunostomy tube; GT: gastrostomy tube

Dates	Care team	Key findings	Plan and interventions
January 2021	Primary care	Increased agitation. Self-injurious behavior Broken teeth	Referral to dental clinic. Care coordination between medical and dental teams.
January 2021	Primary care	Presents for pre-procedural oral sedation for dental exam	Administered oral sedation.
January 2021	Dental	Tooth #4 fractured with periapical lesion. Tooth #5 broken with periapical lesion. Tooth #27 broken with active caries. Generalized smooth surface initial carious lesions.	Nitrous Oxide sedation. Oral exam. Application of SDF to initial carious lesions. Referral to OR for treatment. Care coordination between medical and dental teams.
March 2021	Medical/Dental operating room	Need for GJ exchange. Non-restorable teeth #4, 5 and 27.	General anesthesia. IR GJ tube exchange. Oral exam. Adult dental prophylaxis. Extraction #4, 5 and 27.
April 2021	Primary care	Reduced pain. Reduced agitation.	Reduction in frequency of oral sedation medications.
July 2022	Primary care (medical/dental)	Pain and agitation due to dental concerns has returned.	Review of longitudinal goals. Family discussion about full extraction. Referral to dental for operatory care. Care coordination between medical and dental teams.
October 2022	Dental	History and physical performed in preparation for OR.	Plan to extract remaining teeth.
November 2022	Inpatient/Medical/ Dental/ Operating Room	Admitted to hospital with acute illness. Need for GT placement. Non-restorable teeth #6, 7, 21, 25, 28, 29, and 31.	General anesthesia. GT exchange. Oral exam. Ultrasonic scale, hand scale, polish and flossed. Extraction #6, 7, 21, 25, 28, 29, and 31.

## Discussion

Patients with severe neurological conditions such as Rett syndrome have more complex, acute, and chronic needs for medical and dental care [1,3-5]. At the same time, they have more likely to face financial and logistical barriers to care [8,9]. Negative social drivers of health negatively impact outcomes for oral and medical care [9,11,12]. These medical, oral, and social conditions make providing optimal for some patients challenging.

Our patient faced not only medical and dental complexity but also social complexity. Social drivers of health including language, education, and insurance barriers and long waits for specialized dental care, impacted her medical and dental care, as well as her quality of life. To optimize her care, interprofessional collaboration was crucial for success. The interdisciplinary teams across clinic and hospital-based care worked together to overcome financial and logistical barriers to equitable care and our patient’s success illustrates the positive impact of such collaboration for a medically complex patient with significant social drivers of health.

While this patient had been cared for by the medical team since early childhood, the dental team began providing integrated services at the clinic five years prior to this report. Patients cared for in the clinic lacked adequate access to oral health care, so the clinic added four dental operatories to the patient care hallway. The School of Dentistry staffed the clinic with attending dentists and dental students. The integration of the services was key to the success of the care this patient received, because the dental and medical teams were coordinated, and the family had deep trust of the system.

The key to decreasing the patient’s pain and agitation was addressing her dental needs. At the same time ongoing family conversations ensured that her quality of life was optimized. Moderate and general sedation was imperative to dental surgical success, which in turn improved her medical status and quality of life, as well as her family’s quality of life. Coordination between medical and dental teams was essential to decrease risks of repeated anesthesia and surgeries. Due to the patient’s underinsurance, the coordination not only decreased risk, but also decreased financial costs. Clear communication between teams and within the medical record allowed for improved care coordination. As the care of this patient took place in a teaching clinic, an additional long-term benefit of the collaborative approach to care is that future health care professionals, including physicians and dentists, learn firsthand the importance of collaboration.

More consistent access to dental care over the patient’s life might have allowed her to avoid dental abscesses and extensive extractions. However, given the limitations in daily home oral health care related to biting and lack of dental insurance, the collaborative, interdisciplinary approach described above allowed for access to care that aligned with the family’s goals and the highest quality of life for the patient.

## Conclusions

This case study of a young adult with Rett syndrome and severe dental caries demonstrates that collaborative, interprofessional teamwork can help overcome barriers to dental care for medically complex patients. This case offers a specific, interprofessional approach to addressing social drivers of health including: 1) Longitudinal primary care continuity, which helped to facilitate trust and shared decision-making; 2) Longitudinal goals of care conversations; 3) Excellent case management advocacy and coordination of multiple services/surgical procedures; 4) Consistent interpretation use/certified bilingual providers; and 5) Creative collaboration between medical and dental teams housed within a single clinic. This successful approach could be replicated in other interprofessional situations, working with medical and therapy teams. Social drivers of health often impact those with complex medical conditions, interprofessional collaborations in primary care clinics aimed at addressing these issues can improve health.

Creative, interprofessional care in the service of health equity strengthens the healthcare system. This case demonstrates one success representing the possibility for integrated whole-person care, with a focus on the social drivers of health. 
